# Bayesian ages for pollen records since the last glaciation in North America

**DOI:** 10.1038/s41597-019-0182-7

**Published:** 2019-09-24

**Authors:** Yue Wang, Simon J. Goring, Jenny L. McGuire

**Affiliations:** 1 School of Biological Sciences, Georgia Institute of Technology, Atlanta, GA 30332 USA; 2 Department of Geography, University of Wisconsin-Madison, Madison, WI 53706 USA

**Keywords:** Palaeoecology, Biogeography

## Abstract

Terrestrial pollen records are abundant and widely distributed, making them an excellent proxy for past vegetation dynamics. Age-depth models relate pollen samples from sediment cores to a depositional age based on the relationship between sample depth and available chronological controls. Large-scale synthesis of pollen data benefit from consistent treatment of age uncertainties. Generating new age models helps to reduce potential artifacts from legacy age models that used outdated techniques. Traditional age-depth models, often applied for comparative purposes, infer ages by fitting a curve between dated samples. Bacon, based on Bayesian theory, simulates the sediment deposition process, accounting for both variable deposition rates and temporal/spatial autocorrelation of deposition from one sample to another within the core. Bacon provides robust uncertainty estimation across cores with different depositional processes. We use Bacon to estimate pollen sample ages from 554 North American sediment cores. This dataset standardizes age-depth estimations, supporting future large spatial-temporal studies and removes a challenging, computationally-intensive step for scientists interested in questions that integrate across multiple cores.

## Abstract (summary)

**Table:** 

Measurement(s)	age
Technology Type(s)	Bayesian Model
Factor Type(s)	number of age controls • number of pollen samples • interval between age controls
Sample Characteristic - Environment	sediment
Sample Characteristic - Location	North America

Machine-accessible metadata file describing the reported data: 10.6084/m9.figshare.9768113

## Background & Summary

Fossil pollen can be used as a proxy for past vegetation changes, allowing us to infer or compare these changes to past environmental conditions. With pollen we can determine the mechanisms driving ecosystem changes and infer future responses to environmental change. The pollen record of the last 22,000 years, since the Last Glacial Maximum (LGM), is particularly useful because it traverses many abrupt changes in climate, such as Bølling–Allerød warming event (15,000 BP) and Younger Dryas cooling event (12,700 BP)^1^ with high temporal resolution^2^. Many impactful macroecological studies have used long-term pollen data from this period to examine vegetation responses to climate shifts^3,4^, the extent to which no-analog climates result in no-analog communities^5–7^, and the effectiveness of reserve prioritization methods^8,9^. These studies have demonstrated that plant taxa individualistically track late-Quaternary climate rather than shifting simultaneously as communities of species^3,4^. They have also shown that no-analog plant communities existed in the past and will be common in the near future under novel climate^5–7^. Pollen analysis will continue to provide critical insights that inform our predictive models. However, they are currently hampered by the extensive data processing necessary before big-picture questions can be addressed.

The greatest obstacle to vegetation studies that span large spatio-temporal scales is the challenge of establishing accurate, consistent pollen ages. To explore spatio-temporal changes across pollen samples, the events in one pollen record must be linked to events across records. Thus it is critical to construct a consistent and accurate temporal framework across sediment cores (e.g., Giesecke, 2014^10^ and Blois, 2011^11^). In sediment cores, age-depth models estimate pollen sample ages by integrating radiocarbon-dated specimens with pollen sample depths. Several different age-depth model routines have been developed (clam^12^, Bacon^13^, OxCal^14^, etc.) that can produce different age estimations for a single core by incorporating uncertainty and depositional processes differently^15^. Even when using a single age-depth model, the selection of different parameter values can produce different age estimates. Data for largescale pollen studies are most frequently acquired from community data repositories, such as the Neotoma Paleoecology Database (https://www.neotomadb.org/)^16^. Paleoecological databases, including Neotoma, compile pollen records and their estimated ages from authors’ publications and contributions. Thus, if one were to try to use multiple cores for analysis, the cores would likely possess a variety of low-quality age estimations, including poorly-calibrated radiocarbon dates and simple interpolations between dates. Few age estimations would include uncertainty estimations. Any analyses that use pollen records derived from multiple studies must recalculate age-depth models and parameter values for consistency and accuracy^12,17–19^.

Two main classes of age-depth models are used in the literature: classical and Bayesian. Classical age modeling includes linear interpolation, polynomial fits, or smooth splines. These estimated ages can be accurate, but estimated age uncertainties are usually underestimated^18^. Bayesian age-depth models, including Bacon, BChron^20^, and OxCal, have features that can consider the underlying uncertainty of the chronological controls, but many also have the benefit of using other prior information in model construction. For example, Bacon provides support for estimates of accumulation rates and their autocorrelation within the core. As a result, Bayesian age-depth models can increase accuracy when chronological controls are complex and/or possess high resolution sampling^12^. More importantly, the way Bayesian methods accomidate uncertainty removes the subjectivity from deciding which dates should be included^13,21^. Bacon has become an important tool for modelling age-depth relationships in large scale studies^22–25^.

To facilitate continental-scale analyses in North America, we created a single dataset that provides Bacon age estimations for all suitable North American pollen records from Neotoma (as of January 2018). In this dataset, we used advanced age-depth models with consistent priors while considering the full breadth of conditions for core deposition, and we provided appropriate uncertainty estimations for the sample ages. With the publication of this dataset, any researcher interested in using this pollen dataset will have access to accurate, consistent age estimations, allowing them to directly compare records across North America. We anticipate that this dataset will be used extensively by the ecological community to address critical questions about habitat change throughout the Pleistocene, Holocene, and into the present.

## Methods

### Data source

#### Pollen records

**Fig. 1:**
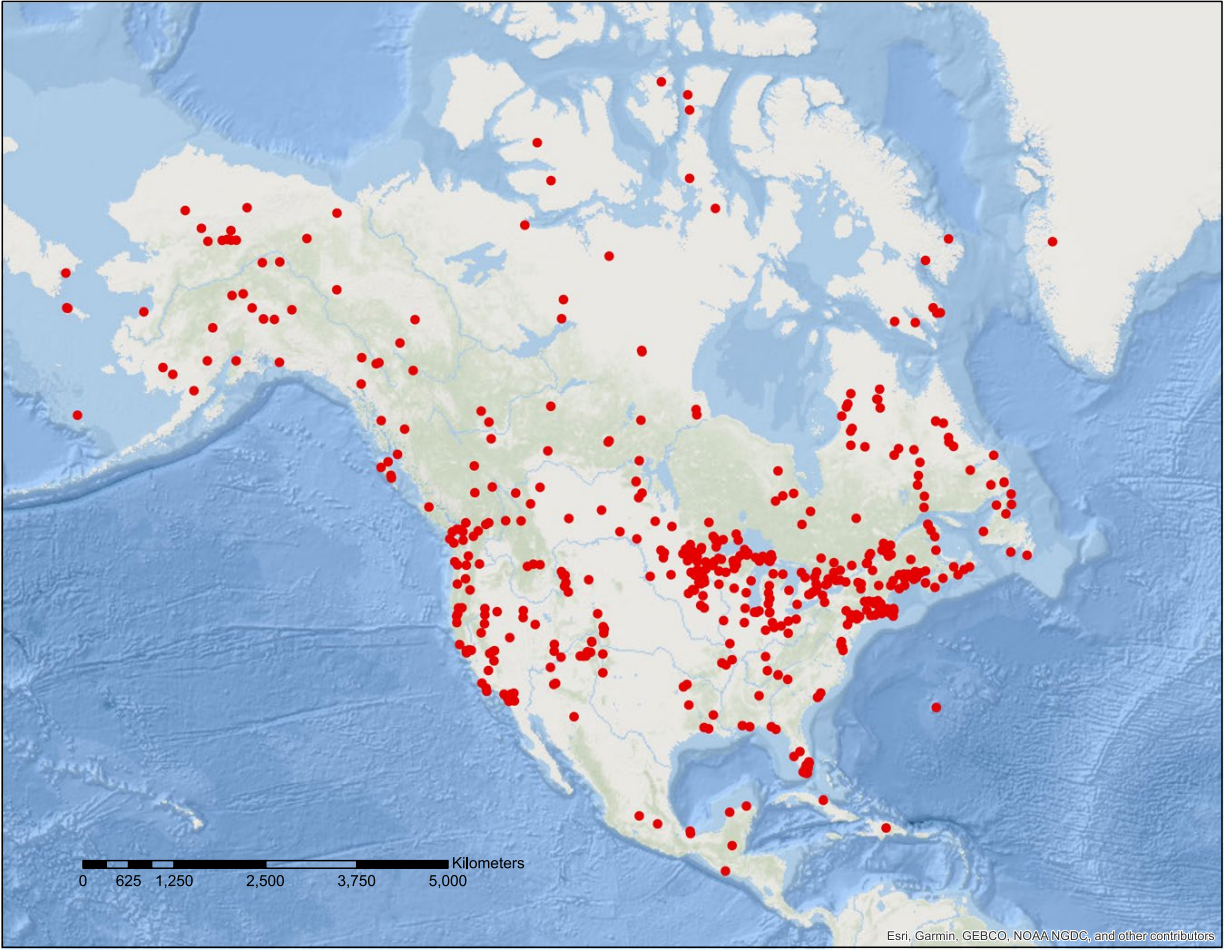
Map of included pollen-containing sediment cores from North America.

We used the neotoma package^26^ for R^27^ to query the Neotoma database for records that met the selection criteria. We got sedimente cores containing 23,187 pollen samples from 531 sites throughout North America (172.25°W to 48.25°W, 10.25°N to 79.75°N) (Fig. 1). The Neotoma database contains over 32,000 datasets from more than 16,000 globally distributed sites, including fossil pollen, vertebrates, diatoms, plant macrofossils, and other types of data from terrestrial paleoecological and paleoenvironmental studies^16^. Pollen data compiled by the Neotoma database is the most complete dataset available for North America.

**Fig. 2:**
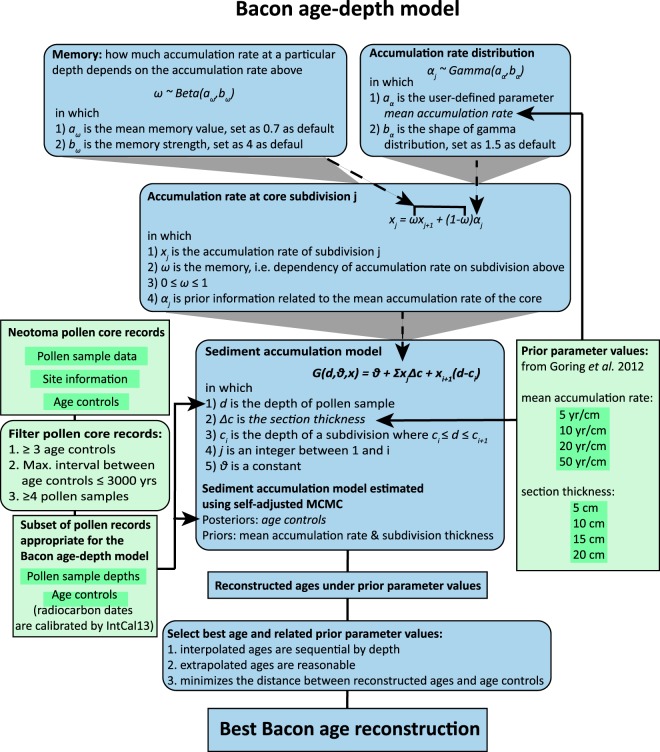
Experiment design. We collected pollen records from Neotoma, built Bacon under different prior parameter values, and selected best estimated age to get the final Bacon age. Rectangular boxes indicate data, and rounded rectangular boxes indicate process. Green boxes indicate data and process in the Neotoma database, and blue boxes indicate data and process in the Bacon age-depth model. Radiocarbon dates are calibrated using IntCal13^38^.

We used three criteria to select pollen-containing sediment cores that could produce accurate Bacon models: (1) At least three age controls (including radiocarbon dates and other age controls from the most recent age estimation in Neotoma) are present in the core; (2) The maximum interval between two adjacent age controls is less than 3000 years; and (3) There are at least four pollen samples in the record, as it is unusual to analyze cores with few pollen samples in vegetation history analyses (Fig. 2). We occasionally included a subset of long cores that met these criteria but excluded the subdivisions with age intervals exceeding 3,000 years.

### Age controls

We used established age controls in Neotoma, including but not limited to radiocarbon dates, U-Pb dates, biostratigraphic events, and the age control at the top layer of the sediment cores if it exists. There are more than one chronology in some sediment cores in Neotoma. These new chronologies have previously been added to the database using more advanced age-depth models, such as the Bacon age model in this work. New chronologies have also been added using updated age controls. For example, Blois *et al*.^11^ added biostratigraphic events as age controls to pollen-containing sediment cores in eastern North America. For the sediment cores that have more than one set of age controls, we used the set that was uploaded to Neotoma most recently.

### Age-depth model

There are four common Bayesian age-depth model frameworks: BChron^20^, Bpeat^28^, OxCal^14^, and Bacon^13^. Of these four, BChron and OxCal are not informed by prior information about sediment accumulation rates, which can lead to models that rely too heavily on chronological controls that may themselves be problematic as a result of secondary processes within the sedimentary basin^13^. OxCal is also particularly suited to chronologies from vertebrate sites that have time-averaged assemblages of taxa rather than regularly-accumulating lake sediment cores. Bpeat assumes a linear accumulation rate within deposits, which is unlikely within natural environments^29,30^. Bacon uses prior information about regional accumulation rates with estimates of the rate of change of accumulation rates to estimate an accumulation rate at each sample depth using a gamma autoregressive process^13^. Studies^13,18^ have demonstrated that Bacon produces the age estimations with the most appropriate uncertainty estimation. We used the rbacon package^31^ in R and bulk-baconizing repository^32^ – a set of codes to implement Bacon code for building age-depth models for many cores simultaneously – to estimate ages for each pollen record^33^.

Bacon uses a gamma autoregressive semiparametric model to simulate sediment deposition processes within subsections of the core^13^. For a given section of the core, Bacon estimates a rate of deposition based on the position of chronological controls within the core, and the rate of deposition of adjacent sections. Users can define prior estimates for accumulation rates, the memory parameter, and can additionally add “breakpoints”, or hiatuses where they believe that these accumulation and memory parameters might have different priors. Age estimates are then generated from the cumulative sum of the deposition rates, as a function of section thickness (which can also be set). Bacon has two advantages over classical models: it does not over-inflate estimated age uncertainties^18^ and it identifies and avoids outlier radiocarbon dates by relying on accumulation rates^13^.

**Fig. 3:**
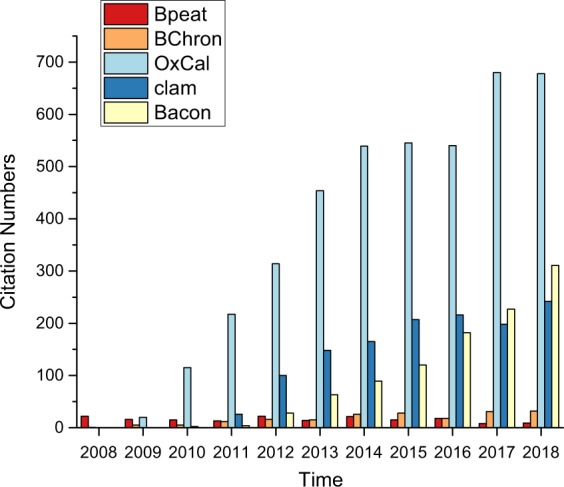
Change in the number of papers using different age-depth models over the last 10 years. The cited works for Bpeat^28^, Bchron^20^, OxCal^14^, clam^12^, and Bacon^13^.

Bacon has been used in 1054 published works according to Google Scholar citations (Fig. 3), getting much more usage than Bpeat and BChron. OxCal is more cited, in part because of a longer history within the discipline, but also because the software is also used to calibrate individual radiocarbon dates, whether or not they are then used in chronology construction. Clam is more cited before 2016, but is less cited in the recent two years. Despite the Bacon’s more complicated implementation, it is nonetheless continuing to increase in usage because of its advantages of higher precision age estimation throughout the core and because training has been widely available.

#### Identifying prior parameter values

Bacon requires the researcher to identify and assess prior information, including mean sedimentation rate and section thickness. These priors can be estimated regionally^29^ or can vary regionally or locally based on prior knowledge. However, Bacon can be highly sensitive to selection^18^ of priors, so a clear framework for the selection of priors should be established. The Bacon model divides a core into equal-length sections. Section length is set by the user, although Bacon may suggest a default section thickness. For each iteration of the model and for each section, an accumulation rate is sampled randomly from a Gamma distribution. The sampled accumulation rate is based on the prior defined for the model run and adjusted using the accumulation rate estimated from the section below. This strategy effectively accounts for autocorrelation of accumulation rates within a core. Accumulation rate sampling minimizes the uncertainty of the fit through the chronological controls. The sampling from the Gamma distribution at a single sample provides a posterior estimate of accumulation for the individual section (as opposed to the prior defined by the user). Using Markov Chain Monte Carlo (MCMC) sampling, the accumulation rate for each section of the core is then estimated once per iteration, across some large number of iterations, resulting in a posterior distribution of accumulation rates for each section. Sample ages can then be estimated by combining the accumulation rates (cm/yr) with the section thicknesses (cm), providing a distribution of ages at any depth within the core.

We ran Bacon for each core using the bulk-baconizing repository^32^, using 16 combinations of prior parameters: four mean accumulation rates and four section thicknesses (Fig. 2). Goring *et al*.^29^ identified likely prior accumulation rates for Holocene sediment cores in the northeastern United States. According to their work, accumulation rates rarely exceed 50 yr/cm and are rarely lower than 5 yr/cm. The median accumulation rate is 10 yr/cm and the mean rate is 20 yr/cm. We proposed four mean accumulation rates in our work based on the Goring *et al*.^29^ paper: 5 yr/cm, 10 yr/cm, 20 yr/cm, and 50 yr/cm. When Bacon approximates the deposition process, sedimentation rate varies smoothly within the core using the gamma model. We also used four values as potential section thickness: 5 cm, 10 cm, 15 cm, and 20 cm. Section thicknesses smaller than 5 cm result in so many core sections that computational processing becomes unwieldy; section thicknesses larger than 20 cm prevent the model from smoothing sufficiently.

We also set a hiatus in the cores from the Northeast and upper Midwest when European settlement was indicated in the age controls, because accumulation rate increased significantly after European settlement^34,35^. This pattern of increasing accumulation rates is apparent in many cores across eastern North America and may be due to a number of factors, including increasing erosion, but is also due to sediment compaction and de-watering with depth and basin dynamics^36^. Dawson *et al*.^35^ listed 185 cores including a hiatus of European settlement from Neotoma. These cores that had identified settlement horizons that were assigned a hiatus (of length 10 yr, the minimum hiatus length in Bacon). This allowed us to apply a different accumulation rate prior to and after European settlement in the Northeast and upper Midwest.

#### Selecting the best prior parameter values and estimated ages

We established a set of rules by which we could consistently select the best priors and age estimation results for all analyzed pollen records (Fig. 2). Once Bacon has been run, each pollen sample possesses an estimated age along with an uncertainty estimation. These estimated ages were only calculated for subdivisions of the cores that contained sufficient age controls. We compared the results of the 16 models and excluded any results where interpolated ages were not sequential or extrapolated ages were not reasonable, i.e. younger than today (2020 AD, given the uncertainty of estimated ages). We then selected the model with the shortest distance between the estimated ages and the age controls. As the method we used here is a dot-to-dot fit and flawed, we also visually checked the 16 age-depth models for each sediment core to confirm that our selection is the best. All the age estimations that we produced list the prior mean accumulation rate and section thickness used in Bacon^33^.

## Data Records

All new age reconstructions have been uploaded to the relevant pollen core record in the Neotoma database, and a full dataset can be downloaded from figshare^33^. See Online-only Table 1 for a list of all data.

## Technical Validation

### Comparing Bayesian age estimations with previous age estimation in Neotoma

Neotoma provides an extensive amount of data, including high-quality and low-quality age estimations. Some age-depth models only used simplistic interpolations between dates. Some age estimations are radiocarbon dates without calibration. Many age estimations are simply calibrated using a look-up table in Neotoma without using a proper calibration curve. Most age-depth models did not give uncertainty estimations. For the 554 sediment cores in this work, 202 sediment cores do not have properly-calibrated ages, 162 sediment cores only have estimated ages from a simplistic interpolation between chronological controls, and 275 sediment cores do not have uncertainty estimations for sample ages. 240 out of 554 sediment cores in this work have properly-calibrated ages from age-depth models with sample age uncertainty estimation.

**Fig. 4:**
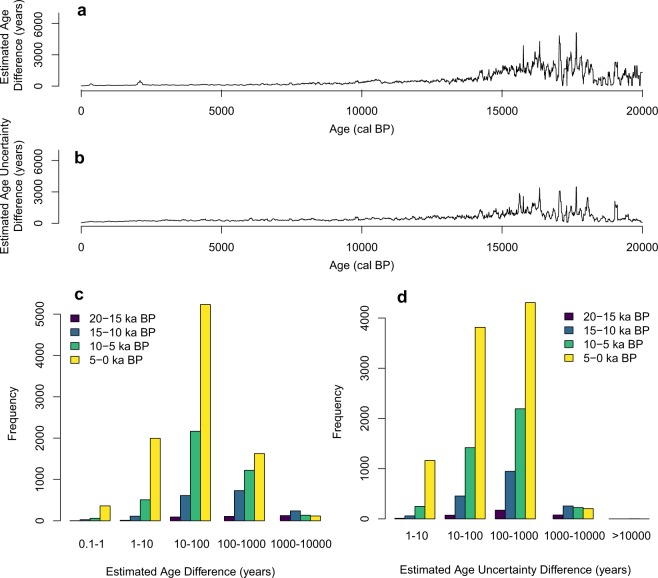
Comparison between Bayesian ages in this work and previously estimated ages in Neotoma for 240 sediment cores, all cores that have calibrated ages. (**a**,**b**) Are average differences of estimated age and age uncertainty between Bacon ages from this work and previous Neotoma age-depth models. We calculated differences every 10 years and averaged them over 100-year-long intervals. (**c**,**d**) Are frequency distributions of the data shown in (**a**,**b**), respectively. Each age-depth model was subdivided into 5000-year-long segments (colors) to visualize the distribution of records of different ages.

We plotted all the available age estimations against the Bayesian age from this work in each pollen record^33^. For 314 of the 554 sediment cores, this work is the first to provide a properly-calibrated age estimation with uncertainty using an established age-depth model. For the other 240 sediment cores that already have good age estimations, the dataset in this work provides an additional age estimation with reliable uncertainty using a Bayesian age-depth model. Most pollen samples are younger than 10,000 cal BP (Fig. 4c,d). Differences between Bayesian ages and previously-estimated ages generally range from 10–100 years (Fig. 4c). Differences between Bayesian age uncertainty and previously-estimated age uncertainty generally ranges from 100–1000 years (Fig. 4d). The Bayesian ages estimated in this work and previous age estimations increasingly diverge, particularly after 15,000 cal BP (Fig. 4a,b). Sample ages older than 15,000 cal BP are typically extrapolated by traditional age-depth models. The extrapolated age estimations are sensitive to parameter settings in the age-depth model. Moreover, this dataset provides a consistent temporal framework for large spatio-temporal analysis covering 554 sediment cores across North America, avoiding errors by individual age estimations at each pollen record.

## Usage Notes

The estimated ages are expressed as calendar years before present (cal BP), in which ‘present’ is 1950 AD. The uncertainty is estimated with 95% confidence, i.e. the min and max values represent 2.5% and 97.5% values in quartiles. Depth indicates the position of each pollen sample from the top of the core.

In this work, the Bacon age models do not consider possibilities of a hiatus in the core deposition other than the European settlement hiatus. Sudden changes in the deposition environment, such as flooding events or human settlement, may result in an abrupt shift in the mean accumulation rate. Accumulation rate is not necessarily correlated before and after a hiatus. Bacon provides methods for estimating ages when there is known hiatus in core deposition, but the depth where hiatus occurs and a possible shift in accumulation rate must be decided for that core specifically.

This work uses established chronological controls in Neotoma. However, the established chronological controls may be problematic. For example, core top and biostratigraphic events are common chronological controls in age-depth models, but some of those chronological controls are recorded in Neotoma without uncertainty estimation, which is required by the Bacon age-depth model. We used 2 years as uncertainty if the chronological controls from Neotoma do not have uncertainty estimation, but it is a rough estimation and may underestimate the uncertainty of sample ages. Another potential source of error is hard water effects in the radiocarbon chronological controls that were dated in early periods. Dissolved carbonate in freshwater can cause a dilution of ^14^C in the freshwater reservoir and result in older-than-real radiocarbon dates. Reservoir corrections have been applied to those chronological controls, but not all the corrections are recorded in Neotoma. Age-depth models based on those uncorrected radiocarbon dates will produce errors in the age estimation. Future work should look up the record and the related publications core by core manually to correct the problematic chronological controls.

We have provided the output of 554 Neotoma pollen-containing sediment cores that meet our criteria for possessing a subdivision with sufficient age controls for accurate age estimations and reliable uncertainty estimations. If the reader wishes to integrate additional pollen-containing sediment cores, new Bacon age estimations can be calculated using the same consistent criteria using the scripts provided in association with this work. Estimated ages, together with prior parameters, can be sent to the author of this paper for addition to the dataset download and/or an appropriate Neotoma data steward for database upload (https://www.neotomadb.org/contacts/investigators). The framework and scripts in this work can also be applied to other regions (such as European pollen records) and other paleo periods. Readers may need to change the prior values based on the deposition environment of sediment cores they are interested in.

## Data Availability

The script used to compile pollen records, run Bacon, and select the best age estimations for each pollen record is available in Github repository https://github.com/yuewangpaleo/BaconAgeNeotoma. The script “BaconAgeCode.R” was run using R^27^. Necessary packages and repository for this code include: bulk-baconizing^32^, rbacon^31^, neotoma^26^, and Bchron^37^.

## Online-only Table

**Online-only Table 1:** List of all data files archived in the repository.

Data Type	Data Content	Variables/Files		Description	Unit	File Name	Data
Excel	core data (complete core has Bacon age)	site ID		core site ID in Neotoma	—	siteinfo_fullcore.csv	https://doi.org/10.6084/m9.figshare.c.4385825
		site name		site name	—		
		longitude		negative from 0 to -180: Western Hemisphere	degree		
		latitude		positive from 0 to 90:Northern Hemisphere	degree		
		dataset ID		core ID in Neotoma	—		
		handle		core name	—		
		acc.mean.old		mean accumulation rate through the complete core	yr/cm		
		acc.shape.old		gamma distribution shape	—		
		thick		thickness of each section	cm		
		mem.strength		accumulation autocorrelation parameter	—		
		mem.mean		mean value of memory	—		
		hiatus		if European settlement hiatus is applied	—		
		acc.mean.mod		mean accumulation rate after European settlement	yr/cm		
		acc.shape.mod		gamma distribution shape after European settlement	—		
		reliableold		the oldest age that is reliable; i.e., the oldest age control	yr BP		
		reliableyoung		the youngest age that is reliable; i.e., the youngest age control	yr BP		
		notes_selection		no good age: the top ages are inappropriately extrapolated; no order age: there are inverse ages through the core depths	—		
		description		description of sites from Neotoma	—		
Excel	core data (a part of core has Bacon age)	site ID		core site ID in Neotoma	—	siteinfo_partcore.csv	https://doi.org/10.6084/m9.figshare.c.4385825
		site name		site name	—		
		longitude		negative from 0 to -180: Western Hemisphere	degree		
		latitude		positive from 0 to 90: Northern Hemisphere	degree		
		dataset ID		core ID in Neotoma	—		
		handle		core name	—		
		top depth		top depth in the core that has Bacon age	cm		
		bottom depth		bottom depth in the core that has Bacon age	cm		
		acc.mean.old		mean accumulation rate through the complete core	yr/cm		
		acc.shape.old		gamma distribution shape	—		
		thick		thickness of each section	cm		
		mem.strength		accumulation autocorrelation parameter	—		
		mem.mean		mean value of memory	—		
		hiatus		if European settlement hiatus is applied	—		
		acc.mean.mod		mean accumulation rate after European settlement	yr/cm		
		acc.shape.mod		gamma distribution shape after European settlement	—		
		description		description of sites from Neotoma	—		
folder (Cores_full.tar; Cores_part.tar)	Bacon age	age control	lab ID	—	—	handle.csv	https://doi.org/10.6084/m9.figshare.c.4385825
			age	dated age of age controls	radiocarbon dates: radiocarbon yr BP		
					non-radiocarbon dates: calendar yr BP		
			error	uncertainty of dated age controls	yr		
			depth	age controls depth	cm		
			cc	corresponding number to distinguish date types: 0 for calendar dates; 1 for Northern Hemisphere terrestrial radiocarbon dates	—		
		age per iteration in MCMC	—	each column is reconstructed age in each iteration in MCMC	yr	handle_number of sections_priorout.csv	
		commands	—	chosen commands in Bacon to produce age-depth model	—	handle_number of sections.bacon	
		parameter values	—	parameter values that make up the age-depth model for each MCMC iteration	—	handle_number of sections.out	
		age-depth graph	—	age-depth graph	—	handle_number of sections.pdf	
		age	depth	depth of pollen samples	cm	handle_number of sections_ages.txt	
			min	youngest age under 95% confidence range	yr BP		
			max	oldest age under 95% confidence range	yr BP		
			median	median age	yr BP		
			wmean	weighted mean age	yr BP		
		depth control	—	depths that model reconstructs age	cm	handle_depths.txt	
		setting	—	input parameter values to Bacon	—	handle_settings.txt	
folder (Evaluation.tar)	Bacon age evaluation	—		compare all the current available age-depth model age in Neotoma against Bacon age in this work	—	handle.pdf	https://doi.org/10.6084/m9.figshare.c.4385825
